# The Strength of the Nutrient Solution Modulates the Functional Profile of Hydroponically Grown Lettuce in a Genotype-Dependent Manner

**DOI:** 10.3390/foods9091156

**Published:** 2020-08-21

**Authors:** Biancamaria Senizza, Leilei Zhang, Begoña Miras-Moreno, Laura Righetti, Gokhan Zengin, Gunes Ak, Renato Bruni, Luigi Lucini, Maria Isabella Sifola, Christophe El-Nakhel, Giandomenico Corrado, Youssef Rouphael

**Affiliations:** 1 Department for Sustainable Food Process, Università Cattolica del Sacro Cuore, 29122 Piacenza, Italy; biancamaria.senizza@unicatt.it (B.S.); leilei.zhang@unicatt.it (L.Z.); mariabegona.mirasmoreno@unicatt.it (B.M.-M.); 2 Department of Food and Drug, University of Parma, 43124 Parma, Italy; laura.righetti@unipr.it (L.R.); renato.bruni@unipr.it (R.B.); 3 Department of Biology, Science Faculty, Selcuk University, 42130 Konya, Turkey; gokhanzengin@selcuk.edu.tr (G.Z.); akguneselcuk@gmail.com (G.A.); 4 Research Centre for Nutrigenomics and proteomics (PRONUTRIGEN), Università Cattolica del Sacro Cuore, 29122 Piacenza, Italy; 5 Department of Agricultural Sciences, University of Naples Federico II, 80055 Portici, Italy; sifola@unina.it (M.I.S.); christophe.elnakhel@unina.it (C.E.-N.); giandomenico.corrado@unina.it (G.C.)

**Keywords:** *Lactuca sativa*, polyphenols, metabolomics, antioxidants, eustress

## Abstract

Considering that functional components of plant foods are mainly secondary-metabolism products, we investigated the shaping of health-promoting compounds in hydroponically grown butterhead lettuce (*Lactuca sativa* L. var. *capitata*) as a function of the strength of the nutrient solution utilized. To this aim, untargeted metabolomics profiling, in vitro antioxidant capacity (total phenolics, 2,2-diphenyl-1-picrylhydrazyl (DPPH), 2,2′-azino-bis(3-ethylbenzothiazoline-6-sulphonic acid) (ABTS), cupric reducing antioxidant capacity (CUPRAC), and ferric reducing antioxidant power (FRAP) assays), and the inhibition of selected enzyme activities were investigated in two butterhead lettuce cultivars with different pigmentation, i.e., green and red Salanova. Full-strength nutrition, together with half- and quarter-strength solutions of macronutrients, was tested. Our results indicate that by reducing the nutrients strength, we could elicit a distinctive shaping of the phenolic profile of lettuce. It is noteworthy that only specific classes of phenolics (namely, lignans and phenolic acids, followed by flavones and anthocyanins) were modulated by the induction of nutritional eustress (fold-change values in the range between −5 and +11). This indicates that specific responses, rather than a generalized induction of phenolic compounds, could be observed. Nonetheless, a genotype-dependent response could be observed, with the red cultivar being much more responsive to nutritional deprivation than the green Salanova lettuce. Indeed, analysis of variance (ANOVA) confirmed a genotype x nutrition interaction in red Salanova (*p* < 0.001). As a consequence of the changes in phenolic composition, also the antioxidant capacity (*p* < 0.001) and amylase inhibition (*p* < 0.001) properties were affected by the growing conditions. However, the effect on cholinesterase and tyrosinase inhibition was poorly affected by the nutritional strength. Provided that yields are not compromised, the application of a controlled nutritional eustress in hydroponically cultivated lettuce may represent a valuable strategy to produce food with tailored functional features in a sustainable manner.

## 1. Introduction

As autotrophs, plants have evolved sophisticated mechanisms to take up inorganic molecules in solution from the soil [1,2]. Mineral absorption is therefore a selective and efficient process, of fundamental importance in plant physiology. In addition, knowledge in this scientific area is central to sustainable agriculture and environmental protection [3].

In recent years, the development of high-throughput analytic studies of small metabolites (i.e., metabolomics) has represented a major technical breakthrough in plant science [4]. Metabolomics has allowed scientific advances in understanding plants’ response and adaptation to mineral availability [5,6,7], also because “omics” can provide a comprehensive view without any assumption about the levels and the effects of experimental factors [8,9]. Plant nutrition studies are, however, hindered by two factors. Soil is complex, variable, and dynamic in terms of its chemical, physical, hydraulic, and biological characteristics [10]. Moreover, plant mineral uptake and translocation, being strictly dependent on water movement, are highly dependent on environmental conditions, such as temperature and light (e.g., intensity and quality). Because of the plant-soil-atmosphere relationship, the molecular analysis of the effects of mineral nutrition in plants is typically performed in hydroponics. Although the term hydroponics may have different connotations, currently, it is mostly applied to indicate any plant production system using a water-based nutrient solution (NS) without soil [11]. For all these reasons, the response of various crops to a range of chemical stressors, such as mineral deficiency, toxicity, and osmotic potential, has been investigated using hydroponics [12,13,14,15].

Even if different formulations have been created, typically, the amount of mineral elements in the NS is set to the highest possible concentration without incurring toxicity and stress [16,17]. The NS in hydroponics is a manageable and flexible experimental factor useful to understand and modulate a plant’s response to nutrient availability beyond mineral withholding, such as by varying the strength and the ion ratio of the NS [18]. Investigations aiming at elucidating the effects of global NS alterations have also a practical impact [19], considering that hydroponics is increasingly used in the commercial production of several crops (i.e., tomatoes, strawberries, cucumbers, peppers, eggplants, lettuce) [20].

There is a consolidated consensus that an adequate supply of mineral elements is central to crop yield. More recently, growing evidence has indicated that the reduction of nutrients to a level that does not result in deficiency symptoms has metabolic consequences of interest also for applied research [21,22]. Specifically, nutritional eustress activates physiological responses and molecular mechanisms that elicit the accumulation of health-promoting bioactive compounds (i.e., antioxidants) necessary for plant adaptation to suboptimal environments [23].

Evidently, these findings provide an opportunity to meet consumers’ increasing demand of high-quality fresh vegetables associated with health benefits [24]. Indeed, epidemiological studies have shown a correlation between a plant-based diet and nutritional benefits associated with fruits and vegetables rich in phytochemical compounds. Lettuce (*Lactuca sativa* L.) is considered an important source of phytoconstituents such as carotenoids, vitamins, and polyphenols [25]. Although it is already widely consumed, biotic and abiotic elicitations have been effectively applied to increase its phytochemical content and thus its perception as a “healthier” food. Regarding the modulation of microconstituents in growth media, the total flavonoid content was significantly increased following Ca^2+^ supplementation and correlated with the antioxidant activity of lettuces [26]. On the other hand, macronutrients deprivation showed an increase in antioxidant compounds such as ascorbic acid, polyphenols, and carotenoids [27,28,29]. The biosynthesis of these antioxidant metabolites important for human diet, results from the activation of plant defense mechanisms to environmental stresses. Application of chemical elicitors, such as jasmonic acid, elicited the accumulation of phenolic compounds (i.e., flavonoids and phenolic acids) as well as of other phytochemicals such as vitamin C, chlorophylls, and carotenoids [28]. The content of these pigments was also modulated under different light wavelengths [29], with the highest increase induced by fluorescent light plus blue LED and monochromic red radiation. Furthermore, lettuce plants exposed to chilling stress (4 °C for 1 day) rapidly activated key genes involved in the biosynthesis of phenolic compounds, ascorbic acid, and α-tocopherol [30]. From these studies, it is evident that lettuce polyphenol content and, thus, polyphenol intake through the human diet, relevant for the prevention of several diseases, can be modulated by NS management.

Here, we aimed at investigating the changes in lettuce phytochemicals in response to a sub-optimal plant nutrient supply, with a focus on the shaping of phenolic compounds, antioxidant capacity, and inhibition potential towards health-related key enzymes. To test this hypothesis, we used lettuce as a model crop, considering two lettuce genotypes that underlie different leaf colorations. Lettuce is a major crop widely cultivated in hydroponics also because of its rapid growth, abundant edible leaves, and fast production cycle [26,31]. We focused on two similar butterhead lettuce varieties that in normal growing conditions develop red-pigmented and green-pigmented leaves. A factorial experimental design, with three progressively reduced concentrations of macronutrients and an invariant micronutrient supply, was chosen. Furthermore, high-resolution ultra-high performance liquid chromatography-quadrupole time-of-flight (UHPLC-QTOF) mass spectrometry-based untargeted metabolomics was used to comprehensively unravel the impact of the reduction of the NS strength on phytochemical profiles.

## 2. Materials and Methods

### 2.1. Plant Grow Conditions and Experimental Design

The work was carried out on two butterhead lettuce (*L. sativa* L. var. *capitata*) varieties, namely, green and red Salanova^®^ (Rijk Zwaan Italia, Bologna, Italia), grown with a closed Nutrient Film Technique (NFT) in a 28 m^2^ open gas-exchange growth chamber (Model Process-C5, Spagnol srl, Treviso, Italy). The experimental system and the environmental conditions were as previously described [28].

The experimental design was full factorial, with the factor “genotype” (G) having two levels (i.e., green and red Salanova), and the factor “strength of the nutrient solution” (S) having three levels, namely, full strength (FS), half strength (HS), and quarter strength (QS). The concentrations of macronutrients in the FS nutrient solution were: 9.0 mM nitrate, 1.0 mM phosphorous, 4.0 mM potassium, 2.0 mM sulfur, 4.0 mM calcium, 1.0 mM manganese, and 1.0 mM ammonium. The concentrations of the macronutrients in HS and QS nutrient solutions were half and a quarter, respectively, of those of the FS solution. In each nutrient solution, the concentrations of microelements were invariant (i.e., 15 μM iron, 9 μM manganese, 0.3 μM copper, 1.6 μM zinc, 20 μM boron, 0.3 μM molybdenum). The average values of the electrical conductivity of the FS, HS, and QS solutions measured in the NFT channels were 1.5 ± 0.1, 0.75 ± 0.1, and 0.5 ± 0.1 dS m^−1^, respectively.

### 2.2. Sample Preparation

We used a randomized complete-block experimental design with the six treatments and three replicates for each cultivar, making a total of 18 experimental units, comprising 12 plants each (*n* = 216 plants). Determinations of total phenolic and total flavonoid content, antioxidant activity assays, and enzyme inhibitory activities were carried out using lyophilized biomass, whereas metabolomics analysis was done on liquid nitrogen-quenched fresh biomass. In both cases, the 12 plants within each replicate of each treatment were pooled.

### 2.3. Total Phenolic and Total Flavonoid Contents

The total phenolic and flavonoid contents were determined as previously reported [32], using the Folin-Ciocalteu and the AlCl_3_ assays, respectively. Total phenolics were expressed as gallic acid equivalents (mg GAEs/g extract), while total flavonoids as rutin equivalents (mg REs/g extract).

### 2.4. UHPLC-QTOF Mass Spectrometry Profiling

Each lettuce sample (1.0 g) was extracted in triplicate in 20 mL of 80% methanol (LCMS grade, VWR, Milan, Italy), acidified with 1% v/v HCOOH using ultrasounds at the amplitude of 80% for 20 min (Fisher Scientific model FB120, Pittsburgh, PA, USA). The extracts were centrifuged at 8000 *g* for 15 min at 4 °C (Eppendorf 5810R, Hamburg, Germany), and the supernatants were directly filtered into HPLC glass vials through 0.22 μm cellulose syringe filters.

The phenolic profile was investigated in duplicate through ultra-high-pressure liquid chromatography electrospray ionization quadrupole-time-of-flight mass spectrometry (UHPLC-ESI/QTOF-MS) as previously reported [33], with the exception that an Agilent Pursuit 3 PFP column (100 × 2.0 mm, 3 μm) was used for chromatographic separation. Briefly, the positive scan mode was used to acquire monoisotopic accurate masses in the 100–1000 m/z range at a rate of 0.8 spectra/s. The binary gradient separation used a mixture of water and acetonitrile (both with 0.1% HCOOH, both LCMS grade, VWR, Milan, Italy) as mobile phase. Quality controls (QCs) were prepared by pooling aliquots of each sample and then analyzed in data-dependent tandem mass spectrometry. To this aim, 12 precursor ions per scan were selected for auto-MS/MS (absolute threshold = 1000; relative threshold = 0.001%; collision energies = 10, 20 and 40 V). The injection volume was 6 μL, nitrogen was used as both sheath gas (10 L/min and 350 °C) and drying gas (8 L/min and 330 °C), the nozzle voltage was 300 V, and the capillary voltage was 3.5 kV in both MS and MS/MS acquisitions.

Raw data were then processed in Profinder B.07 (from Agilent Technologies, Santa Clara, CA, United States), and annotation was done according to the whole isotope pattern (accurate mass, isotopic spacing, and isotopic ratio), with a mass accuracy of <5 ppm. Mass and retention time alignment, as well as filters by frequency (only those compounds identified within 100% of replications in at least one treatment were retained) were also adopted. The database exported from Phenol-Explorer 3.6 [34] was used as a reference for identification. Based on the Metabolomics Standards Initiative [35], an annotation of level 2 (i.e., putatively annotated compounds) was achieved. QCs were elaborated in MS-DIAL 3.98 (RIKEN Center for Sustainable Resource Science: Metabolome Informatics Research Team, Yokohama, Japan), using MS/MS spectra to confirm features obtained by Profinder B.07 [36]. For this purpose, publicly available MS/MS experimental spectra built in the software (e.g., MoNA, Mass bank of North America) were used.

The phenolic dataset was then semi-quantitatively elaborated [37]. Phenolic compounds were grouped into classes and subclasses, and cumulative intensities were quantified using calibration curves of pure phenolic standards (purity >98%; from Sigma-Aldrich, St. Louis, MO, USA), analyzed under the above-described analytical conditions. Ferulic acid (for hydroxycinnamic acids and other phenolic acids), quercetin (for flavonols), sesamin (furan and furofuran lignans), cyanidin (anthocyanins), catechin (flavanols), luteolin (flavones and other remaining flavonoids), resveratrol (stilbenes), and tyrosol (tyrosols and other polyphenols) were used as representatives of their corresponding classes. A linear regression curve (not weighed and not forced to origin) was built using a concentration range of 0.05–500 ppm, and results were expressed as mg equivalents/100 g dry weight (dw).

### 2.5. Determination of Antioxidant and Enzyme Inhibitory Effects

Antioxidant assays included metal chelating, phosphomolybdenum, ferric reducing antioxidant power (FRAP), cupric reducing antioxidant capacity (CUPRAC), 2,2′-azino-bis(3-ethylbenzothiazoline-6-sulphonic acid) (ABTS), and 2,2-diphenyl-1-picrylhydrazyl (DPPH) activities of the extracts as previously described [38]. Results were expressed as Trolox equivalents, and Ethylenediaminetetraacetic acid (EDTA) was used for metal chelating assays. The possible inhibitory effects of the extracts against acetylcholinesterase (AChE, from electric eel acetylcholinesterase (type-VI-S), EC 3.1.1.7), butyrylcholinesterase (BChE, from horse serum, EC 3.1.1.8)) (by Ellman’s method), α-amylase (from porcine pancreas, EC. 3.2.1.1), α-glucosidase (from *Saccharomyces cerevisiae*, EC. 3.2.1.20), and tyrosinase (from mushroom, EC 1.14.18.1) were evaluated through in vitro bioassays.

The enzyme inhibitory effects were determined as equivalents of kojic acid (KAE) for tyrosinase, of galantamine (GALAE) for AChE and BChE, and of acarbose (ACAE) for α-amylase and α-glucosidase.

### 2.6. Statistical Analysis

PASW Statistics 25.0 (SPSS Inc., Segrate, Italy) was used for the analysis of variance (ANOVA; *p* < 0.05) in semiquantitative values of each representative phenolic class, for enzyme inhibitory assays, and for total flavonoids and total phenolics. Homogenous subclasses were investigated according to the Duncan’s post-hoc test. Correlation coefficients between enzymatic assays and different phenolic subclasses were determined according to Pearson in PASW Statistics 25.0 (*p* = 0.05, two-tailed).

Metabolomics raw data were processed in Mass Profiler Professional B.12.06 (Agilent Technologies, Santa Clara, CA, United States) for mass and retention time alignment, for filtering by abundance and by frequency, for normalization and baselining, as previously reported [39]. Thereafter, a fold-change-based hierarchical cluster analysis (Euclidean distance, Ward’s linkage) was carried out for unsupervised distribution. The dataset was next exported into SIMCA 16 (Umetrics, Malmo, Sweden) for orthogonal projection to latent structures discriminant analysis (OPLS-DA) supervised modelling. Outliers were investigated according to Hotelling’s T2 (95% and 99% confidence limits for suspect and strong outliers, respectively; Supplementary Figure S1). CV-ANOVA (*p* < 0.01) cross-validation and permutation testing (for overfitting, N = 200) were also done from the OPLS-DA model. Thereafter, model fitness parameters (goodness-of-fit, R^2^Y, and goodness-of-prediction, Q^2^Y) were recorded, and the variable importance in projection (VIP) analysis was used to identify the most discriminant compounds (VIP score > 1.1) in lettuce grown under different conditions.

## 3. Results

### 3.1. Phenolic Profile of Salanova Lettuce

The polyphenolic composition of lettuce was explored using a metabolomics approach, resulting in the putative annotation of 327 phenolic compounds belonging to different classes. A wide diversity of phenolics could by annotated by our untargeted metabolomics analysis. In particular, 163 flavonoids (including flavanones, anthocyanins, flavonols, and flavanols), 69 phenolic acids, 67 low-molecular-weight phenolics, 19 lignans, and 9 stilbenes were found. The dataset, including composite mass spectrum and ontology classification (i.e., classes and subclasses) of annotated compounds, is provided as Supplementary Material (Supplementary Table S1 for MS annotations and Supplementary Table S2 for MS/MS-identified compounds, respectively).

A strong influence of genetic background could be observed. Indeed, the red cultivar featured a higher content of polyphenols, compared to the green one. The most abundant phenolic classes were lignans, tyrosols, and phenolic acids (Table 1). In particular, low-molecular-weight phenolics (expressed as tyrosol equivalents) reached the highest amounts under quarter-strength growth, with 829 and 1316 mg/100 g eq., followed by lignans compound with 765 and 1682 mg/100 g eq. and phenolics acids with 368 and 876 mg/100 g eq. in green and red Salanova lettuce, respectively.

Salanova lettuce was found to be a rich source of flavonoids, including flavones (i.e., 121–222 and 150–264 mg/100 g eq., in green and red Salanova, respectively) and flavonols (i.e., 54–73 and 85–119 mg/100 g eq., in green and red Salanova, respectively). The less abundant phenolic classes were stilbenes, flavanols, and anthocyanins. Anthocyanins concentration ranged from 23 to 29 mg/100 g eq. and from 27 to 53 mg/100 g eq. in green and red Salanova lettuce, respectively. Cyanidin and pelargonidin derivatives were the most represented phenolics among anthocyanins.

Apart from the genotype, polyphenols content was influenced by the different growing treatments. The HS and QS reduced-nutrient solutions increased the accumulation of polyphenols compared to the full-strength solution. In particular, the highest increase was observed for lignans in red Salanova (+41%). A significant interaction between the red cultivar and the HS treatment was recorded for most of the phenolics classes (anthocyanins, flavones, flavonols, lignans, and phenolic acids). Cultivation in QS nutrient solution increased phenol concentration with respect to FS but not to HS.

Several lignan compounds could be annotated through our untargeted profiling approach, such as episesamin, conidendrin, pinoresinol, arctigenin, 7-oxomatairesinol, isohydroxymatairesinol, medioresinol, dimethylmatairesinol. Regarding phenolic acids, Salanova lettuce was mainly characterized by hydroxycinnamic acids such as chicoric acid, p-coumaric acid, caffeic acid, ferulic acid 4-*O*-glucoside. Low-molecular-weight phenolics were the most relevant phenolics in Salanova lettuce, enriched of subclasses such as alkylphenols, phenolic terpenes, hydroxybenzaldehydes, and hydroxyphenylpropenes. However, flavone compounds (like naringin 4′-*O*-glucoside, tetramethylscutellarein, apigenin 7-*O*-glucoside, luteolin 7-*O*-glucuronide) and flavonols (kaempferol 3-*O*-glucoside, quercetin 3-*O*-glucoside, myricetin 3-*O*-glucoside) were also represented.

**Figure 1:**
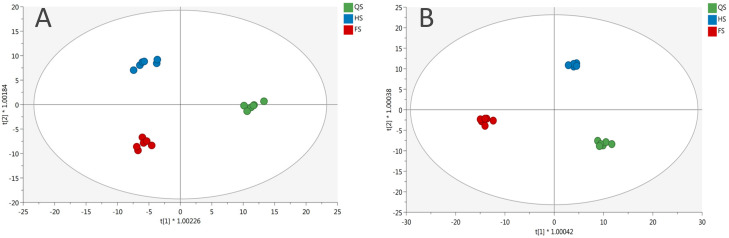
Orthogonal projection to latent structures discriminant analysis **(**OPLS-DA) built using (**A**) green and (**B**) red Salanova samples grown at reduced nutrient concentrations (full strength (FS), half strength (HS), and quarter strength (QS)).

**Figure 2:**
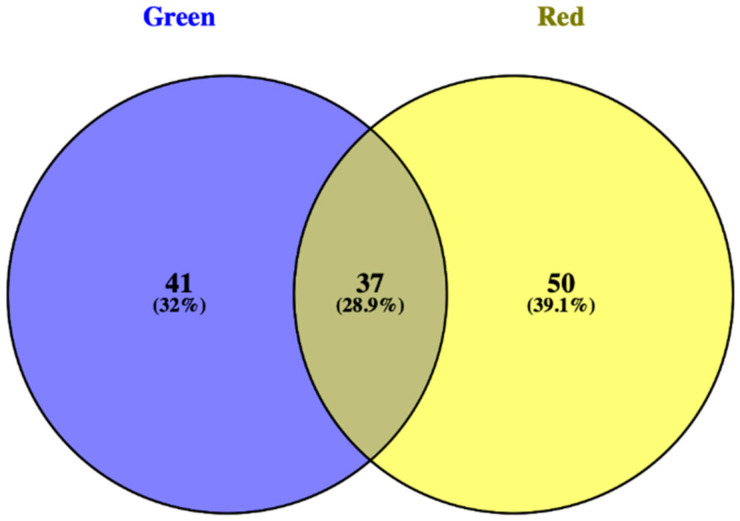
Venn analysis of variable importance in projection (VIP) compound (VIP > 1.1) common between red and green Salanova cultivars.

From each supervised model (Figure 1), the most discriminating compounds were selected using the VIP approach with a threshold of >1.1 (Supplementary Table S3) followed by Veen analysis (Figure 2). The plant response to reduced nutrient concentrations appeared to be strongly influenced by the genotype, since only 28.9% of significant metabolites were common to both cultivars.

### 3.2. In Vitro Antioxidant Capacity

In this study, the in vitro antioxidant properties of both Salanova lettuce cultivars were assessed by different complementary methods, namely, the phosphomolybdenum assay, ABTS [2,2′-azinobis-(3-ethylbenzothiazoline-6-sulfonate)] and DPPH (2,2-diphenyl-1-picrylhydrazyl) scavenging capacity assays, CUPRAC and FRAP methods, and metal chelating activity assay (Table 2).

The half- and quarter-strength nutrient solutions increased the antioxidant activity compared to the full-strength solution. Notably, the red Salanova cultivar grown in half-strength nutrient solution showed two-fold higher values of scavenging ability, compared to the control. In particular, DPPH and ABTS assays recorded values of 32.46 mg TE/g and 41.73, respectively, whilst ferric reduction activity and cupric capacity showed values of 34.87 mg TE/g and 91.40 mg TE/g, respectively. Besides, the control sample was characterized by values of 9.53 mg TE/g and 21.14 mg TE/g for DPPH and ABTS, respectively, whilst ferric and cupric reduction activity values were 19.01 mg TE/g and 49.05 mg TE/g, respectively.

Turning to the green cultivar, different results were obtained. In fact, the quarter-strength solution reported the higher reducing power, especially when assessing cupric and ferric reduction activities (CUPRAC: 32.74 mg TE/g and FRAP: 14.45 mg TE/g), which were 1.2 times higher than those of the control FS solution (CUPRAC: 28.90 mg TE/g FRAP: 11.71 mg TE/g). Regarding the metal chelating activity, the HS solution recorded a 1.2-fold higher value (16.83 mg EDTAE/g) when compared to the FS solution (14.92 mg TE/g).

### 3.3. Enzyme Inhibitory Activity

In addition to the in vitro antioxidant capacity, cholinesterases (AChE and BChE), tyrosinase, α-amylase, and β-glucosidase inhibitory activities were evaluated for both red and green Salanova genotypes (Table 3). Our results revealed that the extracts showed moderate enzyme inhibitory activities, with differences as a function of the strength of the nutrient solution. In particular, the FS solution demonstrated activity against AChE (2.23 mg GALAE/g), while the HS solution was the most effective BChe inhibitor (6.16 mg GALAE/g) in red lettuce. Besides, the QS solution exhibited inhibition against glucosidase (0.81 mmol ACAE/g). Regarding green Salanova, a significant interaction between the genotype and the nutrient solution was recorded with respect to BChE. A significant increase was observed for cultivation in the HS solution compared to the FS solution, while the inhibitory activity did not increase by further decreasing the nutrients (QS solution).

Regarding the red-pigmented lettuce, the HS solution was the most effective AChE inhibitor (1.90 mg GALAE/g), while the full-strength nutrient solution revealed higher potential against butyrylcholinesterase (5.20 mg GALAE/g) and tyrosinase, recording a value of 70.17 mg KAE/g, i.e., 10-fold higher when compared with the other activities. In addition, a significant interaction between the two experimental factors was recorded with respect to amylase for the HS treatment.

### 3.4. Pearson’s Correlation Analysis

Aiming to inspect the contributions of each different class of phenolics to the biological activities we measured, Pearson’s correlations coefficients were investigated (Supplementary Table S4). The most significant correlations (*p* < 0.01) were recorded between flavonols, flavonols, and phenolic acids and both DPPH values (0.74, 0.61, and 0.88 respectively) and ABTS values (0.68, 0.59, and 0.73). In addition, flavonols were correlated with the FRAP assay recorded value of 0.62. Regarding the in vitro enzymatic inhibition, we found a correlation between tyrosinase activity and flavonols (0.81) and phenolic acids (0.69) and a significant (*p* < 0.01) negative correlation between stilbenes and glucosidase activity (−0.62). A lack of correlation between polyphenols content and acethylcholinesterase inhibition and BChE activity was observed. Overall, more interesting results were found considering the red-pigmented lettuce cultivar. In fact, strong correlations (*p* < 0.01) were outlined between phenolic acids and tyrosols by the DPPH, ABTS, CUPRAC, and FRAP assays, and between flavones and metal chelating activity (0.65). Regarding flavonols, a correlation was outlined by DPPH (0.70) and ABTS (0.59) assays, thus confirming the role of polyphenols as the main contributors to the antioxidant properties of this plant food. Finally, no correlations between enzyme activity and polyphenols were detected, except for a negative correlation between butyrylcholinesterase and lignans (0.74).

## 4. Discussion

Lettuce is one of the most important salad vegetables, known as a rich source of vitamins, polyphenols, and antioxidant compounds. The most represented antioxidant compounds are polyphenols, whose concentration varies depending on environmental and genetic factors.

Because of the high content of bioactive compounds, including polyphenols, vegetable-rich diets have been associated to a low risk of chronic diseases, thus entailing strategies aimed at enriching the functional content of vegetables. Hydroponic cultivation has emerged as a promising tool to produce vegetables in a more sustainable and economically valuable manner [40,41,42]. It is noteworthy that hydroponic cultivation offers the possibility to precisely manage crop nutrients availability, hence opening the possibility to modulate the actual functional profile of the produce. In this study, two Salanova cultivars (i.e., green and red) and three levels of nutrients in solution were considered, aiming to assess the impact of nutrient concentration and composition on lettuce growth and bioactive compounds production, as a source of functional foods.

It is well known that most functional compounds, being plant secondary metabolites, result from the interaction between a genetic background and the environment [43,44,45]. Despite using two rather related cultivars, our results from the semi-quantitative analysis of polyphenols profile highlighted the strong influence of the genetic background on the phenolic profile. In particular, the red genotype showed a higher phenolic content compared to the green one. In general, the red genotype resulted more responsive to the applied treatments, in agreement with previous studies [27,32]. Nonetheless, each genotype exhibited a distinctive response to the decrease in nutrients strength, with the red cultivar exhibiting a strength-dependent increase in phenolic acids, lignans, and flavanols. Previously reported literature [46] suggested that the phenolic content is significantly influenced by cultivars, in addition to environmental stressor, which is in accordance with our findings. Polyphenols are abundant micronutrients in our diet, and evidence for their role in the prevention of degenerative diseases such as cancer and cardiovascular diseases is emerging [47]. However, their health-promoting effects depend on the amount consumed as well as on their bioavailability. Interestingly, we did not observe a generalized stress-induced induction, but metabolomic profiling indicated that the amounts of specific phenolic classes (lignans, flavones, anthocyanins, and phenolic acids, Table 1) increased. These low-molecular-weight phenolics have shown high bioaccessibility in simulated gastrointestinal digestion. In particular, plant lignans like sesamin are rapidily absorbed and are detected in the systemic circulation within a few hours from ingestion [48,49]. Furthermore, specific active metabolites are produced from lignans degradation by the colonic microflora, namely, the enterolignans enterolactone and enterodiol, and may have either agonistic or antagonistic effects on estrogens [50,51]. The content of this class of phytoestrogens was strongly increased under nutritional stress in red Salanova, in a stress-dependent manner. Therefore, this nutritional chemical eustress potentially represents a valuable tool to elicit the plant’s metabolic responses leading to a higher accumulation of lignans. Apart from their role in ameliorating menopausal symptoms and their consequences [52], lignans guard against the accumulation of reactive oxygen species, whose overproduction can damage cellular constituents, and play a role in the pathogenesis of different disorders [53]. Indeed, the results we obtained from the radical scavenging assays indicated that the red Salanova HS extracts possessed a significant free-radical scavenging activity, with the highest significant correlations recorded between lignans and DPPH.

Cultivation of red Salanova in HS nutrient solutions also triggered the accumulation of flavonols, with kaempferol-glucoside, quercetin-glucoside, and myricetin-glucoside detected as the major metabolites. These metabolites are known to provide a strong antioxidant effect and can increase superoxide dismutase, catalase, and glutathione peroxidase activities. Accordingly, we outlined a positive correlation between flavonols and free-radical scavenging activity. According to the literature, the antioxidant activity of flavonoids is mainly related to their ability to act as hydrogen donors and efficient scavengers of free radicals from lipid peroxidation [54]. Furthermore [55,56], the absorption of these glycosides has been reported to occur in the small intestine, and its efficiency is higher than that for the aglycone itself.

The low concentration of anthocyanins we found in lettuce (including the red variety) might indicate a major contribution of lipophilic pigments, such as carotenoids, in leaf pigmentation. Indeed, we previously reported *β*-cryptoxanthin, violaxanthin, neoxanthin, lutein, and *β*-carotene as major carotenoids in red Salanova lettuce (ranging 614–1011 μg/g dw) compared to the non-pigmented genotype (ranging 289–444 μg/g dw) [27,32]. In our experimental conditions, the red genotype showed higher in vitro antioxidant activity, likely due to the elicitation of bioactive secondary metabolites like polyphenols, ascorbic acid, and caffeic acid derivatives, as previously investigated [27].

The half-strength nutrient solution was also found to increase α-amylase-inhibiting properties, compared to the full-strength solution, in red Salanova. This enzyme is involved in the digestion of carbohydrates, and its inhibition may represent a strategy to lower the levels of postprandial glycemia via the control of starch breakdown [57,58]. A previous study highlighted the role of the lettuce carotenoid lactucaxanthin as an α-amylase and α-glucosidase inhibitor [59], suggesting its nutritional relevance in the treatment of type 2 diabetes and obesity. On the other hand, both Salanova genotypes, irrespective of the nutritional treatment, showed moderate tyrosinase-inhibiting properties. This key enzyme catalyzes browning and melanin synthesis and has been proposed to manage hyperpigmentation [60].

Although the cultivars chosen were related to one another, their phenolic signatures were distinctive, with phenolic classes differing between the two cultivars (*p* < 0.01). Such genotype-related differences were maintained in response to nutritional stress, with elicitation of specific phenolic classes in each cultivar. The phenolic profile of the red genotype showed significant modulation for anthocyanins, lignans, flavonols, and phenolic acids (*p* < 0.001) in a stress-dependent manner. This is expected if we consider that, under the term phenolics, we include a rather broad diversity of compounds with only a partial sharing of their biosynthetic pathways. Consistently with our metabolomic profiling, colorimetric assays (total phenolics and in vitro antioxidant capacity) and amylase inhibition were significantly affected by the “genotype x nutrients strength” interaction (*p* < 0.01). Pearson’s correlations evidenced that total phenolic content justified a large part of the antioxidant capacity, both in terms of radical scavenging and reducing power assays (correlations 0.79–0.96) and in terms of amylase inhibition (correlation 0.87). Nonetheless, individual phenolic classes showed a comparatively much lower degree of correlation to antioxidant capacity.

It is worthy to note that green Salanova presented some interesting features as well, having the highest content of total flavonoids and the highest inhibitory activity against BChE. Such features were cultivar-dependent and did not show a relationship with the strength of nutrient solution, except for an increase in BChE inhibition in half-strength nutrient solution. The inhibition of AChE and BChE is considered one of the possible therapeutic strategy against neurodegenerative disorders such as Alzheimer’s disease [61], senile dementia, and ataxia. Recently, secondary metabolites from plants such as flavonoids and terpenoids have been proven to possess AChE and tyrosinase inhibitory activities [62,63,64,65,66]. Accordingly, green Salanova displayed higher total flavonoid content compared to the pigmented type, suggesting the putative role of these compounds in modulating cognitive functions.

Considering the mechanisms underlying the shaping of phenolic compounds in plants, it is known that vegetative growth is mainly supported under favorable conditions, while secondary metabolism receives metabolic allocation priority in resource-limited environments [63]. As an example, phenylalanine (a rate-limiting precursor of all phenylpropanoids) is also an essential amino acid for protein synthesis. Notwithstanding, the carbon-nitrogen balance hypothesis [64] suggests that carbon-based secondary metabolites (including phenolics) inversely correlate with nitrogen availability, whereas nitrogen-containing secondary metabolites (e.g., alkaloids) typically show a direct correlation. As reviewed by Heimler et al. [63], the increase in phenolics under low-nitrogen conditions is not generalized and involves specific classes of phenolics. Besides confirming this last point, here we show that nutrients availability modulates phenolics content (in turn modulating functional properties related to phenolics) in a genotype-dependent manner.

## 5. Conclusions

Our results indicate that by reducing the content of macroelements in the nutrient solution, we could significantly modulate the phenolic profile of our lettuce and, therefore, also its antioxidant capacity and amylase inhibition properties. It is noteworthy that the red cultivar was much more responsive to nutritional deprivation, compared to green Salanova lettuce. Furthermore, it is important to notice that only specific classes of phenolics (including lignans, flavones, anthocyanins, and phenolic acids) were shaped by the induction of nutritional eustress, indicating that specific responses should be considered, rather than focusing on a generalized modulation of phenolic compounds’ production. The modulation of nutritional strength did not significantly affect cholinesterase and tyrosinase inhibition; on the other hand, such activities depend on several other phytochemicals in addition to phenolic compounds.

The application of a controlled nutritional eustress in hydroponically cultivated lettuce may represent a valuable strategy to produce food with tailored functional features. Interestingly, the approach proposed can also meet both the current need for an increased sustainability of agricultural production and the demand for healthy foods at consumer’s level.

## Figures and Tables

**Table 1:** Semi-quantitative profile of phenolic compounds in green and red Salanova lettuce (S) in nutrient solutions with different macrocations concentration (C). Results are cumulative untargeted-metabolomics profiling data per each phenolic subclass.

Source of Variance	Anthocyanins	Flavanols	Flavones	Flavonols	Lignans	Tyrosols	Phenolic Acids	Stilbenes
	Cyanidin eq. (mg/100 g)	Catchin eq. (mg/100 g)	Luteolin eq. (mg/100 g)	Quercetin eq. (mg/100 g)	Sesamin eq. (mg/100 g)	Tyrosols eq. (mg/100 g)	Ferulic eq. (mg/100 g)	Resveratrol eq. (mg/100 g)
Salanova (S)								
Green	26.18 ± 1.23 b	31.21 ± 1.83 b	143.5 ± 9.49 b	62.66 ± 2.85 b	724 ± 20.05 b	759 ± 22.66 b	328.2 ± 14.22 b	34.21 ± 2.37 b
Red	45.61 ± 2.37 a	49.61 ± 4.70 a	193.8 ± 13.19 a	106.5 ± 4.77 a	1116 ± 109.51 a	1227 ± 44.80 a	778.0 ± 29.06 a	58.22 ± 5.40 a
Concentration (C)								
Full strength	32.52 ± 1.63	35.18 ± 2.74 b	153.0 ± 14.18 b	72.00 ± 5.30 b	717 ± 37.71 b	897 ± 66.19 b	492.0 ± 51.78 b	48.30 ± 9.39
Half strength	37.16 ± 3.96	36.19 ± 6.57 b	194.0 ± 19.22 a	85.76 ± 9.55 a	820 ± 54.44 b	1022 ± 96.57 a	544.7 ± 81.41 b	43.02 ± 3.27
Quarter strength	38.01 ± 4.72	49.85 ± 4.38 a	159.0 ± 11.17 b	95.99 ± 7.55 a	1224 ± 147.49 a	1059 ± 76.23 a	622.5 ± 78.29 a	47.32 ± 4.45
C × S								
Green × Full strength	29.27 ± 2.15 bc	28.04 ± 2.53	121.0 ± 6.40 c	58.06 ± 5.48 c	729 ± 48.87 c	718 ± 47.40	329.2 ± 21.76 c	30.77 ± 1.54
Green × Half strength	25.97 ± 2.10 c	27.11 ± 2.55	141.4 ± 17.00 bc	56.68 ± 1.72 c	679 ± 29.54 c	728 ± 31.51	286.5 ± 6.08 c	36.12 ± 5.16
Green × Quarter strength	23.29 ± 1.67 c	38.47 ± 2.30	168.1 ± 19.08 bc	73.23 ± 4.05 b	765 ± 9.67 c	830 ± 21.59	368.8 ± 29.56 c	35.72 ± 4.97
Red × Full strength	35.76 ± 1.72 b	42.32 ± 2.52	185.1 ± 20.83 b	85.94 ± 4.01 b	704 ± 61.70 c	1077 ± 64.24	654.7 ± 27.10 b	65.83 ± 16.20
Red × Half strength	48.35 ± 3.80 a	45.27 ± 12.26	246.6 ± 15.14 a	114.8 ± 7.75 a	962 ± 64.65 b	1316 ± 73.80	803.0 ± 49.45 a	49.92 ± 1.12
Red × Quarter strength	52.73 ± 2.95 a	61.23 ± 5.24	149.8 ± 12.29 bc	118.8 ± 5.20 a	1682 ± 107.47 a	1288 ± 63.68	876.3 ± 18.27 a	58.92 ± 2.92
Significance								
Salanova (S)	***	***	**	***	***	***	***	***
Concentration (C)	ns	*	*	***	***	*	***	ns
S × C	***	ns	**	*	***	ns	**	ns

Data are mean ± standard error; *n* = 6. The symbols ns, *, **, and *** indicate a nonsignificant or a significant statistical difference at *p* ≤ 0.05, 0.01, and 0.001, respectively. For each variable, letters indicate statisticallly homogenous groups according to Duncan’s multiple-range test (*p* = 0.05). The effects of the factor Salanova were compared according to Student’s *t*-test.

**Table 2:** Total phenolic acids, total flavonoid content, and antioxidant activities in green and red Salanova lettuce (S) in nutrient solutions with different macrocations concentration (C). GAE, gallic acid equivalents, RE, rutin equivalents, TE, trolox equivalents, EDTAE, ethylenediaminetetraacetic acid equivalents.

Source of Variance	Total Phenolic Content	Total Flavonoid Content	DPPH	ABTS	CUPRAC	FRAP	Phosphomolydenum	Metal Chelating
	(mg GAE)	(mg RE)	(mg TE)	(mg TE)	(mg TE)	(mg TE)	(mmol TE)	(mg EDTAE)
Salanova (S)								
Green	12.41 ± 0.28 b	22.17 ± 1.39 a	3.31 ± 0.86 b	12.28 ± 0.81 b	30.41 ± 1.47 b	12.73 ± 0.58 b	0.74 ± 0.02 b	15.61 ± 0.57 b
Red	22.31 ± 1.42 a	12.80 ± 1.80 b	22.64 ± 2.78 a	30.94 ± 2.34 a	69.33 ± 5.31 a	27.30 ± 2.00 a	1.11 ± 0.05 a	19.52 ± 0.54 a
Concentration (C)								
Full strength	14.87 ± 0.88 b	21.18 ± 1.57 a	5.71 ± 1.30 b	16.73 ± 1.70 c	38.98 ± 3.35 c	15.36 ± 1.17 b	0.85 ± 0.04 b	16.91 ± 0.88 b
Half strength	20.67 ± 2.75 a	14.16 ± 3.02 b	17.08 ± 5.04 a	26.45 ± 4.83 a	60.49 ± 10.23 a	23.46 ± 3.82 a	1.02 ± 0.10 a	19.36 ± 0.93 a
Quarter strength	16.55 ± 1.25 b	17.11 ± 2.04 ab	16.14 ± 3.26 a	21.64 ± 2.74 b	50.14 ± 6.05 b	21.23 ± 2.33 a	0.91 ± 0.07 b	16.42 ± 0.61 b
C × S								
Green × Full strength	12.12 ± 0.39 c	23.07 ± 0.59 a	1.89 ± 0.64 d	12.33 ± 2.10 d	28.90 ± 2.09 d	11.71 ± 0.55 d	0.79 ± 0.05 cd	14.92 ± 1.02
Green × Half strength	12.57 ± 0.57 c	23.90 ± 1.46 a	1.70 ± 0.23 d	11.18 ± 0.36 d	29.58 ± 1.95 d	12.04 ± 0.44 d	0.74 ± 0.02 cd	16.83 ± 1.01
Green × Quarter strength	12.54 ± 0.56 c	19.54 ± 3.89 ab	6.36 ± 2.07 cd	13.34 ± 1.30 d	32.74 ± 3.51 d	14.45 ± 1.42 cd	0.69 ± 0.05 d	15.08 ± 0.89
Red × Full strength	17.61 ± 0.49 b	19.30 ± 3.02 ab	9.53 ± 1.08 c	21.14 ± 0.74 c	49.05 ± 2.10 c	19.01 ± 0.58 c	0.90 ± 0.05 c	18.91 ± 0.88
Red × Half strength	28.77 ± 2.57 a	4.42 ± 0.10 c	32.46 ± 4.16 a	41.73 ± 3.00 a	91.41 ± 8.64 a	34.87 ± 3.43 a	1.30 ± 0.09 a	21.89 ± 0.47
Red × Quarter strength	20.55 ± 0.44 b	14.67 ± 0.84 b	25.92 ± 2.07 b	29.95 ± 1.95 b	67.53 ± 5.28 b	28.01 ± 1.84 b	1.12 ± 0.03 b	17.77 ± 0.36
Significance								
Salanova (S)	***	***	***	***	***	***	***	***
Concentration (C)	***	*	***	***	***	***	***	**
S × C	***	**	***	***	***	***	***	ns

Data are mean ± standard error; *n* = 6. The symbols ns, *, **, and *** indicate a nonsignificant or a significant statistical difference at *p* ≤ 0.05, 0.01, and 0.001, respectively. For each variable, letters indicate statistical homogenous groups according to Duncan’s multiple-range test (*p* = 0.05). The effects of factor Salanova were compared according to Student’s *t*-test.

**Table 3:** Enzyme inhibitory activities in green and red Salanova lettuce (S) in nutrient solutions with different macrocations concentration (C). AChE, acetylcholinesterase, BChE, butyrylcholinesterase, GALAE, galantamine equivalents, KAE, kojic acid equivalents, ACAE, acarbose equivalents.

Source of Variance	AChE	BChE	Tyrosinase	Amylase	Glucosidase
	(mg GALAE)	(mg GALAE)	(mg KAE)	(mmol ACAE)	(mmol ACAE)
Salanova (S)					
Green	2.20 ± 0.14	5.58 ± 0.21 a	64.77 ± 0.90	0.35 ± 0.01	0.79 ± 0.01
Red	1.88 ± 0.21	4.55 ± 0.20 b	65.82 ± 1.37	0.35 ± 0.01	0.84 ± 0.04
Concentration (C)					
Full strength	1.98 ± 0.19	5.25 ± 0.12 a	66.62 ± 1.45	0.35 ± 0.01 ab	0.83 ± 0.02
Half strength	2.16 ± 0.25	5.36 ± 0.32 a	62.54 ± 1.47	0.37 ± 0.02 a	0.85 ± 0.04
Quarter strength	1.98 ± 0.20	4.59 ± 0.34 b	66.72 ± 1.00	0.33 ± 0.01 b	0.78 ± 0.04
C × S					
Green × Full strength	2.23 ± 0.12	5.30 ± 0.19 ab	63.08 ± 1.29	0.35 ± 0.02 b	0.79 ± 0.02
Green × Half strength	2.55 ± 0.05	6.16 ± 0.40 a	63.85 ± 0.65	0.34 ± 0.01 b	0.78 ± 0.02
Green × Quarter strength	1.79 ± 0.39	5.27 ± 0.36 ab	67.38 ± 2.00	0.35 ± 0.02 b	0.81 ± 0.02
Red × Full strength	1.23 ± 0.04	5.20 ± 0.15 ab	70.17 ± 1.59	0.35 ± 0.01 b	0.86 ± 0.02
Red × Half strength	1.90 ± 0.40	4.56 ± 0.18 bc	61.23 ± 2.90	0.40 ± 0.02 a	0.92 ± 0.07
Red × Quarter strength	2.18 ± 0.04	3.90 ± 0.44 c	66.06 ± 0.46	0.31 ± 0.01 b	0.74 ± 0.09
Significance					
Salanova (S)	ns	***	ns	ns	ns
Concentration (C)	ns	**	ns	***	ns
S × C	ns	*	ns	***	ns

All data are expressed as mean ± s.e; *n* = 6. The symbol ns, *, ** and *** indicate a nonsignificant or a significant statistical difference at *p* ≤ 0.05, 0.01, and 0.001, respectively. For each variable, letters indicate statistical homogenous groups according to Duncan’s multiple-range test (*p* = 0.05). The factor Salavanova was compared according to Student’s *t*-test.

## Supplementary Materials

The following are available online at https://www.mdpi.com/2304-8158/9/9/1156/s1, Table S1: Whole dataset produced from untargeted metabolomics analysis carried out in red and green Salanova subjected to different concentrations of nutrient solution. Compounds are presented with individual intensities and with composite mass spectra. Table S2: List of metabolites confirmed with their fragmentation pattern. Table S3: Statistically significant metabolites (VIP > 1.1) common to both green and red Salanova subjected to different concentrations of nutrient solution. Table S4: Correlation matrix (Pearson) showing correlation coefficients computed for the polyphenol classes, antioxidant capacity, and enzyme activity inhibition. Figure S1: Supervised model validation and outliers check. Permutation test outcome and analysis of outliers for A) green and B) red Salanova subjected to different concentrations of nutrient solution.
