# Supplementary material for: The Strength of the Nutrient Solution Modulates the Functional Profile of Hydroponically Grown Lettuce in a Genotype-Dependent Manner
Source: Foods. 2020 Aug 21;9(9):1156. doi: 10.3390/foods9091156 (PMC7555578; doi:10.3390/foods9091156)
Supplement: Supplementary file 1 [file foods-09-01156-s001.zip › foods-891657-supplementary/foods-891657 supple proof.docx]

**Supplementary Material for:**

The strength of the nutrient solution modulates the functional profile of hydroponically grown lettuce in a genotype-dependent manner

Biancamaria Senizza^1§^, Leilei Zhang^1§^, Begoña Miras-Moreno^1^, Laura Righetti^2^, Gokhan Zengin^3^, Gunes Ak^3^, Renato Bruni^2^, Luigi Lucini^1,4^*, Maria Isabella Sifola^5^, Christophe El-Nakhel^5^, Giandomenico Corrado^5^, and Youssef Rouphael^5,^*

^1^ *Università Cattolica del Sacro Cuore, Department for sustainable food process, Piacenza, Italy*; [biancamaria.senizza@unicatt.it](mailto:biancamaria.senizza@unicatt.it) (B.M.S.); [leilei.zhang@unicatt.it](mailto:leilei.zhang@unicatt.it) (L.Z.); [mariabegona.mirasmoreno@unicatt.it](mailto:mariabegona.mirasmoreno@unicatt.it) (M.B.M.M.); [luigi.lucini@unicatt.it](mailto:luigi.lucini@unicatt.it) (L.L.)

^2^ *Department of Food and Drug, University of Parma, 43124, Parma, Italy*; [laura.righetti@unipr.it](mailto:laura.righetti@unipr.it) (L.R.); [renato.bruni@unipr.it](mailto:renato.bruni@unipr.it) (R.B.)

^3^ *Selcuk University, Science Faculty, Department of Biology, Campus, 42130 Konya, Turkey;* [gokhanzengin@selcuk.edu.tr](mailto:gokhanzengin@selcuk.edu.tr) (G.Z.); [akguneselcuk@gmail.com](mailto:akguneselcuk@gmail.com) (A.G.)

^4^  *Università Cattolica del Sacro Cuore, Research Centre for Nutrigenomics and proteomics (PRONUTRIGEN), Piacenza, Italy;* [luigi.lucini@unicatt.it](mailto:luigi.lucini@unicatt.it) (L.L.)

^5^ *Department of Agricultural Sciences, University of Naples Federico II, 80055 Portici, Italy;* [christophe.elnakhel@unina.it](mailto:christophe.elnakhel@unina.it) (C.E.-N.); [sifola@unina.it](mailto:sifola@unina.it) (M.I.S.);[giandomenico.corrado@unina.it](mailto:giandomenico.corrado@unina.it); (G.C.) [youssef.rouphael@unina.it](mailto:youssef.rouphael@unina.it) (Y.R.)

**^§^** These authors contributed equally

***** Corresponding author: [luigi.lucini@unicatt.it](mailto:luigi.lucini@unicatt.it) (L.L.) and [youssef.rouphael@unina.it](mailto:youssef.rouphael@unina.it) (Y.R.)


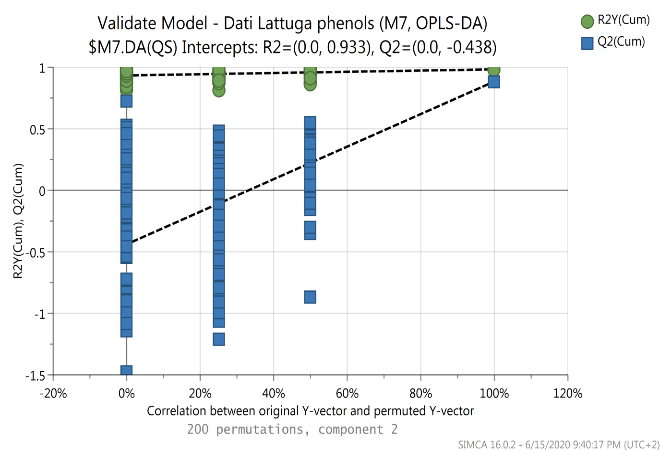

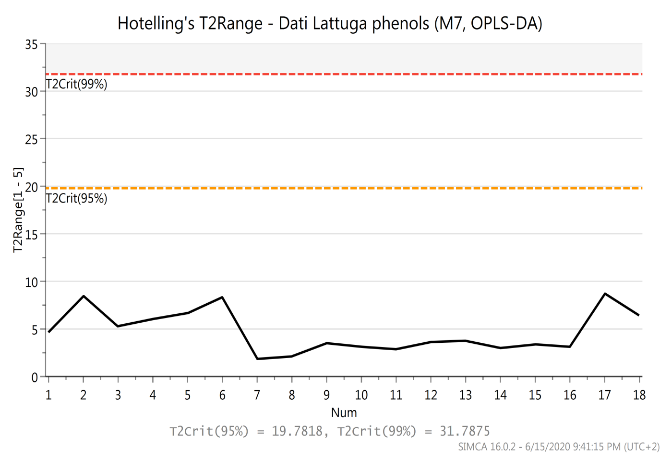


**A**


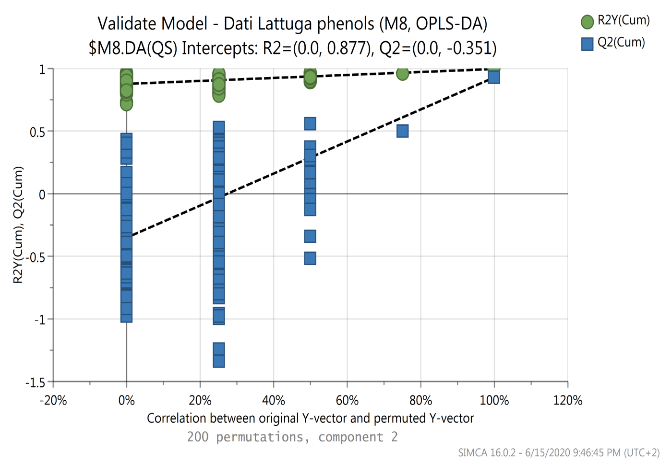

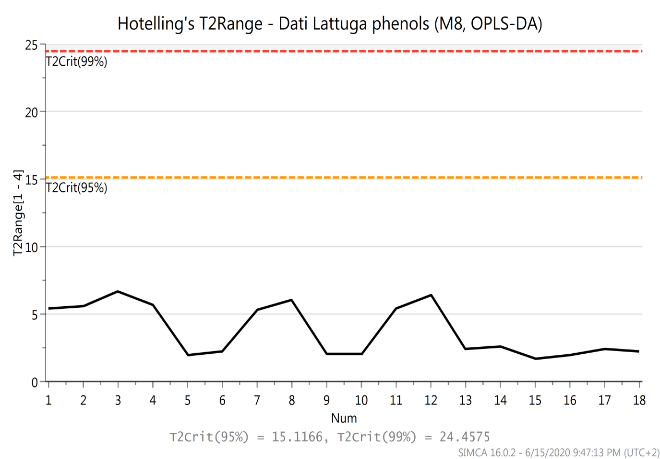


**B**

**Figure S1.** Supervised model validation and outliers check. Permutation test outcome and analysis of outliers for A) green and B) red Salanova subjected to different concentrations of nutrient solution.

**Supplementary Tables:**

**Table S1.** Whole dataset produced from untargeted metabolomics analysis carried out in red and green Salanova subjected to different concentrations of nutrient solution. Compounds are presented with individual intensities and with composite mass spectra.

**Table S2.** List of metabolites confirmed with their fragmentation pattern.

**Table S3.** Statistically significant metabolites (VIP > 1.1) common to both green and red Salanova subjected to different concentrations of nutrient solution.

**Table S4.** Correlation matrix (Pearson) showing correlation coefficients computed for the polyphenol classes, antioxidant capacity, and enzyme activity inhibition.
